# COVID-19-related sickness absence among 4,721 NHS staff in England and its relation with long COVID symptoms: findings from NHS CHECK

**DOI:** 10.1186/s12913-025-13442-w

**Published:** 2025-09-30

**Authors:** B. Dempsey, H. A. Blake, I. Madan, S. A. M. Stevelink, N. Greenberg, R. Raine, A. M. Rafferty, R. Bhundia, S. Wessely, Danielle Lamb

**Affiliations:** 1 Department of Primary Care and Population Health, University College London, London, UK; 2 Guy’s and St Thomas NHS Foundation Trust, London, UK; 3 Department of Psychological Medicine, Institute of Psychiatry, Psychology & Neuroscience, King’s College London, London, UK; 4 Florence Nightingale Faculty of Nursing, Midwifery and Palliative Care, King’s College London, London, UK

**Keywords:** Sickness absence, Long COVID, COVID-19, Healthcare workers, Health providers

## Abstract

**Background:**

Long COVID (LC) occurs when COVID-19 symptoms continue for 12 + weeks after the onset of infection. LC may have several impacts, including sickness absence. This study explored number of days off work due to COVID-19 infection among healthcare workers (HCWs) and predictors of long-term sickness absence (i.e., 4 + consecutive weeks off work).

**Methods:**

Data were from the NHS CHECK survey and included 4,721 HCWs (19.6% of the cohort) who reported COVID-19-related symptoms and sickness absence at two follow-up periods, approximately 12 and 32 months post-baseline (baseline collected between April 2020 and January 2021). We conducted descriptive analysis to explore COVID-19-related sickness absence at both timepoints and to examine differences depending on whether HCWs self-reported symptoms consistent with LC or an LC diagnosis. We used multi-level logistic regression modelling to explore baseline predictors for reporting long-term sickness absence at follow-up among HCWs who reported LC symptoms.

**Results:**

At 12 months, 89.5% of HCWs attributed sickness absence to a COVID-19 infection, while 84.6% reported the same at 32 months. Median self-reported days off work at both timepoints were higher among HCWs who self-reported LC symptoms (12mo = 14 days (IQR = 10–30), 32mo = 7 days (IQR = 4–14)) compared with HCWs who did not (12mo = 9.5 days (IQR = 3.5–14), 32mo = 5 days (IQR = 2–7)). A similar finding was observed for HCWs who reported a formal diagnosis of LC compared with those who did not. There was a noticeable reduction in COVID-19-related sickness absence between our 12 and 32 month follow-up surveys across all groups. Among HCWs who self-reported LC symptoms, predictors for reporting long-term sickness absence at both timepoints included having a pre-existing respiratory illness and being aged 41–50 years.

**Conclusions:**

We found that reporting LC symptoms or having an LC diagnosis were associated with greater sickness absence among HCWs. The cause of the reduction in reported sickness absence between the timepoints is unclear, though it is most likely due to natural recovery from COVID-19, greater support available to staff with LC or the withdrawal of a special COVID-19 sickness absence payment in 2022. The need to support workers with LC to return and remain in work is still present.

**Supplementary Information:**

The online version contains supplementary material available at 10.1186/s12913-025-13442-w.

## Background

Long COVID (LC) occurs when COVID-19 symptoms persist or continue to develop after the acute infection [1–3] and a variety of definitions have been proposed [4, 5]. The National Institute for Health and Care Excellence (NICE) differentiate between two kinds of LC: ongoing symptomatic COVID-19 (symptoms between 4 and 12 weeks after acute infection) and post COVID-19 syndrome (PCS; symptoms 12 + weeks after acute infection) [1]. PCS has since become a popular definition, and is used by the UK National Health Service (NHS) [6]. Other popular definitions include Post COVID-19 Condition (defined by the World Health Organisation [2]), where symptoms continue or newly develop 3 months after the onset of COVID-19 infection, with these symptoms lasting for at least 2 months with no other explanation. Acknowledging the variety of definitions, and in line with the preference of our patient and public involvement and engagement (PPIE) group, in this paper we use LC to describe any condition where symptoms persist after a COVID-19 infection.

The Office for National Statistics (ONS) estimated that over 1.9 million people in the UK had LC in February 2023, with 1.7 million having symptoms for 12 + weeks (~ 3% of the population and ~ 9% of all confirmed COVID-19 infections) [7]. People with LC may experience a range of symptoms which disrupt their lives, including fatigue, cognitive dysfunction, shortness of breath, and muscle and joint pain [7–9]. The ONS further estimated that of the 1.9 million people presumed to be experiencing LC, 59% would indicate that their condition had negatively impacted their ability to undertake day-to-day activities “a little”, while 20% would say that this ability had been “limited a lot” [7]. The persistence of LC symptoms means that many are delayed returning to work [10]. A Swiss population-based cohort study found that 5.8% of working-age people with LC had not returned to work one year after contracting COVID-19 [11], while a Swedish study of 11,955 people found that 13.3% were unable to return to work from COVID-19-related sickness absence after 3 months, and 9% were unable after 4 months [12]. A UK-based study of ONS data from 206,299 people found that 13.1% of working-age people reporting LC required 4 + consecutive weeks off work over a 20 month period [13]. This study also predicted that approximately 27,000 working-age adults in the UK (95% CI: 6,000–47,000; ~0.5% of the total UK workforce) were inactive in July 2022 due to LC, though we note that the wide confidence interval indicates uncertainty in the true number of people affected by LC [13].

This may be pertinent for healthcare workers (HCWs), given their exposure to COVID-19. In the UK, ONS data has shown that, during the first year of the pandemic, HCWs were estimated at ~ 29% increased risk of contracting COVID-19 compared with non-essential workers [14] and were ~ 9% more likely to report symptoms lasting for 4 + consecutive weeks compared to non-essential workers [15]. This means that higher numbers of HCWs may be experiencing LC compared with those in professions associated with lower infection risk. However, it is unclear how many HCWs are currently unable to work due to LC in the UK, as the NHS do not publish this data [16]. Research suggests that NHS sickness absence rates rose at the beginning of the COVID-19 pandemic in March 2020, with periods of high COVID-19 cases strongly correlated with increased staff absences compared to the 2015–2019 average [17]. From March 2020 until September 2022, NHS staff could receive a COVID-19 sickness absence payment which suspended the terms set out in their contract and allowed them additional time off work at full pay [18]. This was revoked from September 1st 2022, and HCWs who were receiving it reverted to their normal contractual sick pay entitlements thereafter (at a maximum 6 months full pay and 6 months half pay) [19]. We have found little research that has focused on occupational impacts linked with LC among HCWs, with most focusing rather on identifying symptoms and risk factors [5].

To address this paucity, the aim of the current study was to examine COVID-19-related sickness absence among a large sample of HCWs in England. Using data from NHS CHECK, we examined sickness absence at two distinct timepoints (April 2021 to January 2022 and February to May 2023). Additionally, using longitudinal regression modelling, we explored baseline predictors associated with higher self-reported sickness absence at follow-up.

## Methods

### NHS CHECK and data collection

We analysed data from NHS CHECK, a longitudinal survey distributed to all HCWs (clinical and non-clinical), students, and volunteers in 18 NHS Trusts across England [20]. NHS CHECK began in 2020 to study the impact of COVID-19 on the health and wellbeing of NHS staff. Trusts were invited to participate via direct emails to senior leadership teams and purposively selected to offer diversity in geographical location, urban versus rural settings, and acute versus mental health Trusts [20]. The Trusts circulated emails promoting the survey to all eligible staff via existing group emails. Staff support teams/leads, Chief Nursing Officers, Medical Directors, occupational health departments, trade union representatives, and wellbeing hub users were also engaged to promote the study. NHS CHECK was discussed during team briefings, advertised via screen savers on Trust computers, and included in Trust newsletters, news items on Trust intranet websites, and closed social media groups.

Data for NHS CHECK were collected across four waves. Baseline data were collected between April 2020 and January 2021. Follow-up surveys were distributed to participants approximately 6, 12 and 32 months post-baseline. Responses across each wave were linked by participants’ email addresses, allowing for longitudinal exploration. To account for attrition between survey waves, a sub-sample of new participants was recruited at 12 months. In total, the NHS CHECK cohort consists of 24,137 HCWs, including 22,554 who began at baseline and 1,583 at 12 months. A full overview of the NHS CHECK methodology and the information collected in the surveys, please refer to the NHS CHECK protocol [20] and pre-registered protocol for this sub-study [21].

### Outcome – Sickness absence

Collected on the 12 and 32 month follow-up surveys, we have data on the self-reported total number of sick days and episodes of sickness absence related to a COVID-19 infection. At 12 months, participants were asked to report sickness absence related to any past COVID-19 infection, while at 32 months, participants were asked to only report COVID-19-related absence in the preceding year, ensuring both measurements were unique. Number of sick days was our primary outcome for descriptive analyses.

We used long-term sickness absence as our outcome when looking for predictors of sickness absence. To do this, we created a binary variable using the total number of sick days and episodes, and followed NHS policy to define long-term sickness absence as 4 + consecutive weeks off work [22]. We chose to create this variable as we believed it would be difficult to meaningfully apply our findings to practice if number of sick days was the outcome. Most HCWs reported one episode of absence (58.4% at 12 months and 63.3% at 32 months), so were classified as requiring long-term sickness absence if they reported 20 + days off work. For the remainder who reported multiple sickness episodes, we did not have an objective measure if any lasted for 4 + consecutive weeks. Instead, for each additional sickness episode they reported, we incrementally increased the total number of sick days required to meet caseness, ensuring that at least one sickness episode exceeded 20 + days, i.e. those who reported two sickness episodes required a minimum of 39 sick days to meet caseness, those who reported 3 episodes required a minimum of 58 sick days, etc.

### Condition – Long COVID

We define participants as having LC if they reported COVID-19 symptoms lasting for 12 + weeks [1]. At 12 and 32 month follow-up, HCWs were asked to report previous COVID-19 infections and those who reported one were then asked to specify which of 24 common COVID-19 symptoms they experienced and for how long. HCWs were also asked to self-report if they had received a formal LC diagnosis from a healthcare professional. We classified HCWs as having LC if they reported experience of any COVID-19 symptom for 12 + weeks [1], or reported a formal diagnosis of the condition. We created an additional variable for formal diagnosis of the condition to allow us to explore the relationship between formal LC diagnoses and sickness absence. Among HCWs we classified as having LC, the most common symptoms were fatigue (51.4%), difficulty concentrating (36.7%), insomnia (35.2%), depression/anxiety (33.0%), and shortness of breath (28.6%) [23].

### Covariates

Variables were included based on input from our PPIE group, and knowledge of the literature [5, 24] and of the variables available for analysis in the NHS CHECK cohort. Demographic covariates in our analyses included age, sex, ethnicity, relationship status and pre-existing respiratory illnesses (i.e. asthma or chronic obstructive pulmonary disease (COPD)). We included symptoms of common mental disorders, measured by the 12-item general health questionnaire (GHQ-12) [25], and used the recommended cut-off score, where scores ≥ 4 indicate probable common mental disorders [25]. The robustness of the GHQ-12 measure has been established even where response options differ slightly from the original (e.g. in this study, there was a small typographical error in one response option of one GHQ-12 item) [26, 27]^1^. Occupational covariates included job role, perceived support from managers and colleagues, contract type, reported thoughts about leaving the NHS, income, contact with COVID-19 patients, perceived access to personal protective equipment (PPE), and confidence in workplace infection control policies. All covariates were collected in the baseline survey, except for reported thoughts about leaving the NHS, which was collected at both follow-up timepoints.

### Analyses

We pre-registered a statistical analysis protocol on the Open Science Framework [21], and this paper addresses research questions four and five. Using population-level data provided by each participating Trust, the cohort was weighted using a raking algorithm based on the age, sex, ethnicity, and job role profile of the workforce to maximise representativeness. To complete the weighting, missing data were imputed using the kth nearest neighbour algorithm using R v4.0.2 [28]. These imputed data were only used to complete weighting. All percentages in the results have been weighted.

We conducted descriptive statistics on the predictor variables and self-reported sickness absence. During descriptive analyses, we removed two HCWs from the 12 month sample as the number of sickness days they reported were deemed likely errors, as 560 and 817 exceeded the maximum number of days we thought possible for a participant to have acquired by our 12 month follow-up period. When calculating weighted percentages for each predictor, we accounted for the proportion of missing data (since HCWs who did not respond to these questions are still included in subsequent analyses thanks to the use of multiple imputation, as described below). To examine differences in self-reported sickness absence between HCWs who either reported LC symptoms or reported a formal LC diagnosis and those who did not, we conducted Mann Whitney U tests.

To examine predictors of long-term sickness absence, we used regression modelling. We used Multiple Imputation via Chained Equations (MICE) to account for missing data in the baseline predictors. A detailed description of the MICE procedure has been provided elsewhere and is believed to be appropriate in imputing realistic values [23]. Two multi-level, multivariable binary logistic regression models were constructed, with imputed data being pooled using Rubin’s rules. The first model used only outcome data collected at 12 months, while the second used only data collected at 32 months. We included only HCWs who reported LC symptoms in these models, and examined data from both timepoints separately, given the notable change in the proportion of HCWs reporting long-term sickness absence. This contributed to the models being under-powered [21]. The models were multi-level as we grouped participants by Trust to account for clustering. In addition, we included a ‘burden period’ variable in the models, i.e., periods of time at baseline when the reported number of COVID-19 cases and deaths were higher or lower, which likely influenced pressure on HCWs and may have influenced the predictors [20]. Presented odds ratios are adjusted for all variables in the model. As a sensitivity test to examine our categorisation of long-term sickness absence, we re-ran the regression models using data from only those who reported one episode of sickness absence. Data analysis was completed using Stata v.18.5 [29].

### Patient and public involvement and engagement

A patient and public involvement and engagement (PPIE) group was set up as part of this study. The group consisted of 16 HCWs with experience of LC from a range of demographic and professional backgrounds. The first group meeting in May 2023 was to discuss the project aims and analyses plans. At the second meeting in October 2023, we discussed the initial findings.

## Results

### Sample characteristics

Of the 5,246 HCWs who self-reported a previous COVID-19 infection at 12 and 32 month follow-up, 4,721 also self-reported information about sickness absence related to their COVID-19 infection (90.0% of those with a previous COVID-19 infection, and 19.6% of the total NHS CHECK cohort). Table 1 presents demographic information for the total NHS CHECK cohort and the subsamples under investigation in this study, while Table 2 presents occupational information. This includes 2,200 HCWs at 12 months and 2,906 HCWs at 32 months, with 385 HCWs reporting COVID-19-related sickness absence at both timepoints. Of the 4,721 HCWs in the sample for this analysis, 75.1% were female, 81.9% were white, 75.7% were married or lived with a partner, and 83.7% did not report a pre-existing respiratory illness. Just under half of HCWs with a previous COVID-19 infection screened positive for symptoms of probable common mental disorders at baseline. Of occupational factors, 8.1% of the sub-sample were doctors, and the remainder were roughly evenly split between nursing, other clinical, and non-clinical staff. Most HCWs perceived at least moderate support from managers (69.7%) and colleagues (80.3%), 89.4% were on a permanent contract, and 66.5% reported thoughts about leaving the NHS. See Supplementary Tables 1a-b and 2a-b for more detailed breakdown of HCWs’ demographic, health and occupational information by those who did and did not report LC symptoms at each timepoint.

**Table 1:** Demographic and health information of the healthcare workers in NHS CHECK

		Total NHS CHECK sample	All HCWs with a COVID-19 infection	Reported a COVID-19 infection at 12 Months	Reported a COVID-19 infection at 32 months
Variable	Categories	*n* = 24,137 (%)	*n* = 4,721 (%)	*n* = 2,200* (%)	*n* = 2,906* (%)
Sex	Female	19,381 (74.1)	3,823 (75.1)	1,771 (75.2)	2,357 (74.4)
	Male	4,491 (24.9)	871 (24.4)	416 (24.4)	534 (25.1)
	Missing	265 (1.0)	27 (0.5)	13 (0.4)	15 (0.5)
Age in years	30 and younger	4,681 (21.3)	646 (15.6)	370 (18.7)	313 (11.3)
	31–40	5,247 (25.0)	911 (22.5)	446 (22.5)	543 (22.8)
	41–50	6,000 (22.0)	1,343 (25.1)	593 (23.4)	865 (26.9)
	51–60	5,691 (20.7)	1,359 (26.3)	612 (26.4)	875 (27.2)
	61 and older	1,439 (6.5)	283 (6.9)	99 (5.4)	202 (8.4)
	Missing	1,079 (4.5)	179 (3.6)	80 (3.6)	108 (3.4)
Ethnicity	White	20,507 (75.9)	4,225 (81.9)	1,904 (79.6)	2,676 (85.0)
	Black	1,045 (8.0)	130 (5.8)	82 (6.6)	53 (4.6)
	Asian	1,572 (12.1)	203 (8.6)	132 (10.1)	86 (7.0)
	Mixed/Other ethnicity	817 (3.3)	144 (3.3)	74 (3.4)	80 (3.1)
	Missing	196 (0.7)	19 (0.4)	8 (0.3)	11 (0.3)
Relationship status	Single/Divorced	6,304 (26.9)	1,108 (24.0)	518 (23.0)	667 (24.1)
	Married/Cohabitating	17,592 (72.2)	3,591 (75.7)	1,677 (76.9)	2,222 (75.4)
	Missing	241 (0.9)	22 (0.3)	5 (0.1)	17 (0.5)
Probable CMDs**	No (GHQ-12 score < 4)	9,811 (40.9)	1,777 (37.1)	778 (34.6)	1,143 (38.6)
	Yes (GHQ-12 score ≥ 4)	10,970 (45.9)	2,160 (46.2)	978 (45.5)	1,360 (47.8)
	Missing	3,356 (13.2)	784 (16.7)	444 (19.9)	403 (13.6)
Pre-existing respiratory illness**	None	21,338 (89.1)	3,891 (83.7)	1,741 (80.3)	2,460 (86.2)
	Reported asthma/COPD	1,216 (5.0)	267 (4.8)	103 (4.3)	186 (5.5)
	Missing	1,583 (5.9)	563 (11.5)	356 (15.4)	260 (8.3)

n = unweighted frequencies (with weighted percentages). NHS = National Health Service in the United Kingdom; HCWs = healthcare workers; CMDs = common mental disorders; GHQ = General Health Questionnaire; COPD = Chronic Obstructive Pulmonary Disease

*385 participants reported COVID-19-related sickness absence at both timepoints

**These variables were not asked of the replenishment sample, accounting for 1,583 missing responses from the total NHS CHECK sample and 563 from HCWs with a COVID-19 infection (with 356 at 12 months and 260 at 32 months)

**Table 2:** Occupational information of the healthcare workers in NHS CHECK

		Total NHS CHECK sample	All HCWs with a COVID-19 infection	Reported a COVID-19 infection at 12 Months	Reported a COVID-19 infection at 32 months
Variable	Categories	*n* = 24,137 (%)	*n* = 4,721 (%)	*n* = 2,200* (%)	*n* = 2,906* (%)
Job role	Nurse	6,127 (29.7)	1,242 (30.8)	638 (32.8)	713 (29.9)
	Doctor	1,727 (9.8)	309 (8.1)	171 (8.5)	171 (7.9)
	Other clinical	7,338 (32.1)	1,438 (32.8)	720 (34.6)	857 (32.0)
	Non-clinical	8,744 (27.7)	1,700 (27.7)	656 (23.6)	1,145 (30.5)
	Missing	201 (0.7)	32 (0.6)	15 (0.5)	20 (0.7)
Perceived manager support**	No/A little support	3,548 (17.3)	670 (17.0)	316 (16.7)	416 (17.3)
	Moderate/Extreme support	18,158 (73.5)	3,401 (69.7)	1,492 (66.4)	2,174 (72.4)
	Missing	2,431 (9.2)	650 (13.3)	392 (16.9)	316 (10.3)
Perceived colleague support**	No/A little support	1,697 (7.3)	301 (6.6)	119 (5.1)	199 (7.9)
	Moderate/Extreme support	20,034 (83.6)	3,774 (80.3)	1,691 (78.4)	2,393 (81.9)
	Missing	2,406 (9.1)	646 (13.1)	390 (16.5)	314 (10.2)
Contract type	Permanent contract	20,480 (85.3)	4,182 (89.4)	1,928 (88.9)	2,600 (90.1)
	Non-permanent contract	3,386 (13.7)	515 (10.2)	260 (10.7)	290 (9.3)
	Missing	271 (1.0)	24 (0.4)	12 (0.4)	16 (0.6)
Thoughts on leaving the NHS	No	–	921 (18.6)	370 (16.7)	694 (22.6)
	Yes	–	3,163 (66.5)	963 (44.5)	2,125 (74.2)
	Missing	–	637 (14.9)	867 (38.8)	87 (3.2)
Income	NHS Band 5 or below	7,599 (27.7)	1,431 (26.3)	691 (28.4)	855 (24.1)
	NHS Band 6 or above	12,570 (54.0)	2,595 (58.3)	1,150 (55.6)	1,655 (60.7)
	Missing	3,968 (18.3)	695 (15.4)	359 (16.0)	396 (15.2)
Contact with COVID-19 patients**	No contact	8,200 (27.1)	1,394 (22.8)	448 (16.3)	1,022 (26.9)
	Contact	10,309 (51.7)	2,100 (52.9)	1,135 (57.3)	1,178 (50.4)
	Missing	5,628 (21.2)	1,227 (24.3)	617 (26.4)	706 (20.7)
Perceived access to PPE**	Inadequate access	1,709 (8.3)	299 (7.3)	151 (8.0)	178 (6.8)
	Adequate access	17,130 (73.8)	3,234 (70.0)	1,479 (68.6)	2,017 (71.5)
	Non-applicable	2,839 (8.6)	518 (8.9)	169 (6.2)	380 (10.8)
	Missing	2,459 (9.3)	670 (13.8)	401 (17.2)	331 (10.9)
Confidence in infection control**	Inadequate confidence	8,211 (37.7)	1,472 (34.1)	674 (33.6)	906 (33.6)
	Adequate confidence	13,406 (53.0)	2,584 (52.4)	1,132 (49.5)	1,669 (55.8)
	Missing	2,520 (9.3)	665 (13.5)	394 (16.9)	331 (10.6)

*n* = unweighted frequencies (with weighted percentages). NHS = National Health Service in the United Kingdom; HCWs = healthcare workers; NHS Band 5 or below = Annual income of £34,581 or lower; NHS Band 6 or above = Annual income of £35,392 or higher (as of January 2024); PPE = personal protective equipment. Data for all variables (except thoughts on leaving the NHS) were collected at baseline, between April 2020 to January 2021, and will be used to explore predictors of reporting long-term sickness absence. Thoughts on leaving the NHS were collected at 12 and 32 months

*385 participants reported COVID-19-related sickness absence at both timepoints

**These variables were not asked to the replenishment sample, accounting for 1,583 missing responses from the total NHS CHECK sample and 563 from HCWs with a COVID-19 infection (with 356 at 12 months and 260 at 32 months)

### COVID-19-related sickness absence

At 12 months, 1,945 of the 2,200 HCWs (89.5%) reported taking time off work due to a COVID-19 infection, while 2,361 of the 2,906 (84.6%) reported the same at 32 months. Table 3 gives information on sickness absence reported by these HCWs. At both timepoints, Mann-Whitney U tests found very strong evidence that HCWs who reported LC symptoms attributed more days off work to COVID-19 infection compared with HCWs who did not report LC symptoms (*p <* 0.001). Similarly, HCWs with a formal LC diagnosis attributed more days off work to COVID-19 at both timepoints compared to HCWs who did not (*p* < 0.001). At 12 months, 20.8% of HCWs with LC symptoms also reported long-term sickness absence due to COVID-19, with 34% of HCWs with a formal LC diagnosis reporting the same. The proportion of HCWs requiring long-term sickness absence reduced from 12 months to 32 months, as only 4% of HCWs with LC symptoms and 13.3% of HCWs with a formal LC diagnosis required it at 32 months.

**Table 3:** Information on sickness absence of healthcare workers with a previous COVID-19 infection, separated by data collected on the 12 month survey (*n* = 2,200; distributed between April 2021 to January 2022) and the 32 month survey (*n* = 2,906; distributed between February to May 2023)

	12 month survey		32 month survey	
	**HCWs with LC symptoms**	**HCWs without LC symptoms**	**HCWs with LC symptoms**	**HCWs without LC symptoms**
*n* (%)	912 (40.0)	1,288 (60.0)	784 (26.8)	2,122 (73.2)
Days off work due to COVID-19				
Median	14 days*	9.5 days	7 days*	5 days
IQR	(10, 30)	(3.5, 14)	(4, 14)	(2, 7)
Range	(0–390)	(0–90)	(0–365)	(0–56)
Long-term sickness absence, *n* (%)	206 (20.8)	53 (4.4)	36 (4.0)	1 (< 0.1)
	**HCWs with a LC diagnosis**	**HCWs without a LC diagnosis**	**HCWs with a LC diagnosis**	**HCWs without a LC diagnosis**
*n* (%)	184 (7.9)	2,016 (92.1)	207 (7.6)	2,699 (92.4)
Days off work due to COVID-19				
Median	25 days*	10 days	10 days*	5 days
IQR	(15, 60)	(5, 15)	(5, 28)	(2, 9)
Range	(0–300)	(0–390)	(0–365)	(0–90)
Long-term sickness absence, *n* (%)	70 (34.0)	189 (9.1)	34 (13.3)	3 (0.2)

*n* = unweighted frequencies (with weighted percentages); IQR = Inter-quartile range of total number of days of sickness absence attributed to COVID-19 infection; HCWs = healthcare workers; LC = Long COVID (defined here as symptoms lasting for 12 + weeks after the onset of the acute COVID-19 infection); Long-term sickness absence = 4 + consecutive weeks off work due to COVID-19 infection, with the table showing the unweighted frequencies of HCWs that reported taking long-term sickness absence (with weighted percentages)

*Results of Mann Whitney U tests found that HCWs with LC symptoms or a formal LC diagnosis attributed significantly more days off work to COVID-19 compared to HCWs who did not, *p* < 0.001

Of the 2,657 HCWs who reported a COVID-19 infection at 12 months, including those who did not report sickness absence, 1,164 (43.8%) also completed the 32 month survey. Of these, 1,145 HCWs (98.6%) reported if they still worked in the NHS at 32 months, while 992 (85.2%) reported if they were currently absent from work due to ill health (see Table 4). Most HCWs (*n* = 1,113, 95.4%) were still working in the NHS, even if they had moved to a different Trust, while a minority (*n* = 32, 3.2%) reported that they had left and the remainder (*n* = 19; 1.4%) did not respond. There were many reasons for choosing to leave the NHS, with only six reporting that this decision was related to COVID-19. Similarly, the majority were not currently absent from work (*n* = 950, 80.5%). Of those who were off sick (*n* = 45, 3.7%), only nine said their absence was related to COVID-19, LC, or a common LC symptom.

**Table 4:** Information from healthcare workers who completed the NHS CHECK 12 month and 32 month follow-up surveys

	All who had COVID-19 at 12 months	Had COVID-19 but not LC symptoms at 12 months	Had LC symptoms at 12 months	
	*n* = 1,164 (%)	*n* = 713 (%)	*n* = 451 (%)	
**Working in the NHS at 32 months?**				
Yes, in the same Trust	1,064 (91.0)	644 (89.4)	420 (94.2)	
Yes, within a different Trust	49 (4.4)	36 (5.7)	13 (2.4)	
No, left the NHS	32 (3.2)*	20 (3.5)	12 (2.2)	
Missing	19 (1.4)	13 (1.4)	6 (1.2)	
**Currently absent from work due to ill health at 32 months?**				
Yes	41 (3.7)**	18 (2.2)	23 (5.6)	
No	950 (80.5)	593 (81.5)	357 (76.4)	
Missing	173 (15.8)	102 (15.3)	71 (18.0)	

LC = Long COVID (defined as symptoms lasting for 12 + weeks after the onset of COVID-19 infection

*Six of these reported COVID-19 as a reason for leaving the NHS (two who did not have LC symptoms at 12 months and four who did)

**Nine of these reported COVID-19 or LC as a reason for their current absence (five who did not have LC symptoms at 12 months and four who did)

### Predictors of long-term sickness absence

After demonstrating that sickness absence was higher among people who reported LC symptoms, we explored predictors of long-term sickness absence among HCWs with LC symptoms. See Table 5 for the results of these analyses. At 12 months and adjusting for all variables, we found strong evidence (*p* = 0.001) that HCWs with a pre-existing respiratory illness had over 3 times the odds of reporting long-term sickness absence compared to those without (adjusted odds ratio, aOR: 3.45, 95%CI 1.91, 6.23). We found weak evidence (*p* = 0.030) of a similar association (aOR = 2.79) at 32 months. At 12 months, we found strong evidence that HCWs aged 41 and older had between 3 and 5 times the odds of long-term sickness absence compared to those aged 30 and younger (41–50 age group aOR: 3.32, 95%CI 1.47, 7.52; 51–60 age group aOR: 3.96, 95%CI 1.38, 11.34; 61 and older age group aOR: 4.99, 95%CI 1.70, 14.61). At 32 months, however, we only found weak evidence (*p* = 0.048) of an association among HCWs aged 41–50 (aOR: 3.62, 95%CI 1.01, 12.96). At 12 months only, we found moderate evidence (*p* = 0.020) that non-clinical HCWs had lower odds than nursing staff of reporting long-term sickness absence (aOR: 0.43, 95%CI 0.21, 0.86). As a sensitivity test, we reran the model with only HCWs who reported one episode of sickness absence and found that no new statistically significant associations were observed. With the exception of being aged 41–50 at 32 months, we also found strong evidence of the aforementioned predictors being associated with increased odds of requiring COVID-19-related long-term sickness absence, though we note that the aOR increased for all (aOR among non-clinical HCWs at 12 months decreased as the sole association < 1.00).

**Table 5:** Results from the multi-level logistic regression models exploring predictors of long-term sickness absence among HCWs who reported long COVID symptoms on the 12 and 32 month surveys

Variable		Odds of Long-Term Sickness Absence:			
		at 12 months (*n* = 912)		at 32 months (*n* = 784)	
	Categories	aOR	95% CI	aOR	95% CI
Sex	Female (Ref)	1.00	–	1.00	–
	Male	0.96	[0.45, 2.07]	1.23	[0.30, 5.10]
Age in years	30 and younger (Ref)	1.00	–	1.00	–
	31–40	3.97	[0.83, 19.02]	1.49	[0.24, 9.04]
	41–50	3.32**	**[1.47**,** 7.52]**	3.62*	**[1.01**,** 12.96]**
	51–60	3.96*	**[1.38**,** 11.34]**	1.42	[0.16, 12.94]
	61 and older	4.99**	**[1.70**,** 14.61]**	1.87	[0.23, 15.32]
Ethnicity	White (Ref)	1.00	–	1.00	–
	Black	1.12	[0.33, 3.82]	0.46	[0.05, 4.10]
	Asian	0.61	[0.21, 1.79]	0.56	[0.02, 15.79]
	Mixed/Multiple and Other ethnic group	1.65	[0.46, 6.00]	0.65	[0.02, 21.64]
Relationship status	Single/Divorced (Ref)	1.00	–	1.00	–
	Married/Cohabitating	0.73	[0.45, 1.18]	1.04	[0.43, 2.50]
Probable common mental disorders	No (GHQ-12 score < 4; Ref)	1.00	–	1.00	–
	Yes (GHQ-12 score ≥ 4)	0.73	[0.31, 1.71]	1.81	[0.41, 7.94]
Pre-existing respiratory illness	None (Ref)	1.00	–	1.00	–
	Reported Asthma/COPD	3.45**	**[1.91**,** 6.23]**	2.79*	**[1.12**,** 6.93]**
Job role	Nurse (Ref)	1.00	–	1.00	–
	Doctor	0.85	[0.36, 1.99]	1.83	[0.20, 16.90]
	Other clinical	0.55	[0.28, 1.08]	2.47	[0.67, 9.15]
	Non-clinical	0.43*	**[0.21**,** 0.86]**	1.09	[0.33, 3.54]
Perceived manager support	No/A little support (Ref)	1.00	–	1.00	–
	Moderate/Extreme support	0.60	[0.25, 1.43]	1.34	[0.28, 6.47]
Perceived collegial support	No/A little support (Ref)	1.00	–	1.00	–
	Moderate/Extreme support	2.17	[0.65, 7.25]	1.02	[0.34, 3.11]
Contract type	Permanent contract (Ref)	1.00	–	1.00	–
	Non-permanent contract	0.80	[0.22, 2.85]	0.14	[0.01, 1.48]
Reported intention to leave NHS	No thoughts about leaving NHS (Ref)	1.00	–	1.00	–
	Thought about leaving NHS	0.92	[0.48, 1.74]	1.44	[0.36, 5.79]
Income	NHS Band 5 or below (Ref)	1.00	–	1.00	–
	NHS Band 6 or above	0.87	[0.52, 1.45]	2.31	[0.80, 6.71]
Contact with COVID-19 patients	No contact (Ref)	1.00	–	1.00	–
	Contact	0.95	[0.45, 2.02]	1.74	[0.43, 7.09]
Perceived access to personal protective equipment	Perceived inadequate access (Ref)	1.00	–	1.00	–
	Perceived adequate access	1.45	[0.57, 3.68]	0.51	[0.11, 2.41]
	Non-applicable	1.40	[0.50, 3.89]	1.92	[0.22, 16.59]
Confidence in infection control policies	Inadequate confidence (Ref)	1.00	–	1.00	–
	Adequate confidence	0.89	[0.52, 1.52]	1.27	[0.42, 3.83]

The 12 month survey was distributed between April 2021 to January 2022, while the 32 month survey was distributed between February to May 2023. HCWs = healthcare workers; long-term sickness absence = a period of 4 + consecutive weeks off work attributed to a COVID-19 infection; aOR = Adjusted Odds Ratio, adjusted for all variables in the table, as well as burden on NHS when predictor data was collected and Trust; 95% CI = 95% Confidence Intervals for the effect of each variable on the outcome. Due to small sample sizes and perfect prediction at 32 months, the Mixed/Multiple and Other ethnic groups were combined for analysis. *p < 0.05; **p < 0.01

## Discussion

We found that self-reported sickness absence was common among HCWs who reported a COVID-19 infection, with 89.5% reporting any COVID-19-related absence prior to our 12 month timepoint (April 2021 to January 2022) and 84.6% reporting similar at 32 months (February to May 2023). We also found that at both timepoints, median days of sickness absence were higher among HCWs who self-reported LC symptoms (12mo = 14 days (IQR = 10–30), 32mo = 7 days (IQR = 4–14)) compared with HCWs who had COVID-19 but did not experience symptoms for 12 + weeks (12mo = 9.5 days (IQR = 3.5–14), 32mo = 5 days (IQR = 2–7)). A similar result was found among HCWs who self-reported a formal LC diagnosis from a medical professional (12mo = 25 days (IQR = 15–60), 32mo = 10 days (IQR = 5–28)) when compared with those who did not (12mo = 10 days (IQR = 5–15), 32mo = 5 days (IQR = 2–9)). Additionally, there was a noticeable reduction in sickness absence across all groups between our follow-up surveys. Finally, we found that having a pre-existing respiratory illness and being between the ages of 41–50 years were associated with greater odds of long-term sickness absence at both data collection periods.

Given the impacts that LC may have [5, 7, 10], it is unsurprising (yet still important) to document that HCWs either self-reporting LC symptoms or with a formal LC diagnosis attributed significantly more days off work to the condition compared with colleagues who contracted COVID-19 but did not experience prolonged symptoms. Of note, the median number of days off for HCWs without LC symptoms (9.5 days at 12 months and 5 days at 32 months) or a formal diagnosis (10 days at 12 months and 5 days at 32 months) aligns with the number of mandatory self-isolation days imposed in the UK at both timepoints [30], while the median number among HCWs with LC symptoms (14 days at 12 months and 7 days at 32 months) or a formal diagnosis (25 days at 12 months and 10 days at 32 months) exceeds these. A study exploring return to work among HCWs with self-reported LC in Scotland found that having less severe symptoms were predictive of faster return to work, even if HCWs had not fully recovered [31]. Further, HCWs who had returned to work rated support from colleagues, management, their organisation and occupational health services as key [31]. This study was conducted during one of our collection period (12 months), and only included a convenience sample of HCWs in Scotland, so we acknowledge that results may not be generalisable to our research.

Our study also found a noticeable reduction in long-term sickness absence between our two follow-up periods. The proportion of staff with LC symptoms reporting long-term sickness absence fell from 20.8% at 12 months to 4.0% at 32 months, while the proportion of staff with a formal LC diagnosis fell from 34.0 to 13.3%. There may be several reasons for this. Firstly, it may be that more HCWs had recovered from their LC symptoms by 32 months than by 12 months, at least to the point of returning to work. A Swiss population-cohort study found that while the prevalence of LC symptom reporting remained relatively high across 24 month follow-up, the symptom severity and associated health impairment reduced over time [32]. Applying this to our sample, while HCWs still experienced LC symptoms into the 32 month follow-up, it may be that these had a smaller functional impact than at 12 months. Relatedly, increased knowledge about LC at the time of the 32 month follow-up could have meant that HCWs knew more about LC and how to manage symptoms, allowing for faster return to work and greater ability to remain in work. It may also have been that HCWs were afforded more support to return to work by 32 months, e.g. reasonable adjustments, redeployment, or access to OH or dedicated LC healthcare services.

Alternatively, the cessation of the special sickness absence payment while off work with a COVID-19-related illness in September 2022 may have been responsible for more HCWs returning to work by the time of the 32 month survey, possibly before they were ready, rather than face reduced pay or possible termination of employment. The decision to withdraw the special payment was criticized at the time for the potential for people to feel pressured to return to work prematurely [33, 34]. At the same time, the removal of this payment may have motivated HCWs to engage in a return to work and recovery plan, allowing for greater return to work. Our PPIE group shared their own and colleagues’ experiences of returning to work following LC and endorsed these three reasons (i.e., natural recovery, increased knowledge and support, and withdrawal of the special payment) as potential explanations for why long-term sickness absence reduced between both timepoints. The need to support people with LC is still present, and further work is needed. For example, the LOCOMOTION (LOng COvid Multidisciplinary consortium Optimising Treatments and servIces acrOss the NHS) team [35] published resources, identifying barriers and enablers, which can be widely implemented to support people with LC [36, 37].

The majority of HCWs who reported a previous COVID-19 infection at 12 months and completed the 32 month survey were still working for the NHS (95.4%) and were not on sick leave (80.5%). Of the minority that had left the NHS or were currently absent from work, very few named COVID-19 or LC as a contributing reason. Regardless, 56.2% who reported a COVID-19 infection at 12 months did not complete the 32 month follow-up. This included 588 HCWs who reported LC symptoms at 12 months, many of whom may have left the NHS or were absent from work by 32 months. Without knowing more about those who did not respond to the follow-up survey, it is difficult to make more concrete claims about the long-term impact of LC symptoms on HCWs’ occupational outcomes.

We found that being above 41 years of age, having a pre-existing respiratory disorder and being a non-clinical worker were associated with greater odds of reporting COVID-19-related long-term sickness absence at 12 months. At 32 months, there was only weak evidence that being aged between 41 and 50 and having a pre-existing respiratory illness were predictors of COVID-19-related long-term sickness absence. This may be due to the reduction in long-term sickness absence observed between our 12 and 32 month surveys. We also note that the “significant” association for the 41–50 age group at 32 months may be a spurious result due to multiple testing. Regardless, having a pre-existing respiratory illness and being of middle or older age have both been identified as predictors of reporting LC symptoms in other studies [24, 38, 39] and in the NHS CHECK cohort [23]. Moreover, more severe symptoms are likely predictive of extended sickness absence [31]. As having a pre-existing respiratory illness and being older are both predictors of developing LC, it may be that these individuals are more likely to experience severe symptoms, which could in turn delay their return to work. Additionally, LC may exacerbate existing asthma/COPD symptoms, resulting in longer absences, while middle and older age people may require longer to recover from illness compared with younger colleagues, regardless of whether it is COVID-19-related [40]. We also know that risk of developing long-term health conditions increases with age, and it is likely that other co-morbid conditions we did not measure in this study may prolong absence following COVID-19 infection.

### Strengths and limitations of the study

The primary strength of this study was its longitudinal nature, which allowed us to examine predictors of sickness absence using data that were collected at least one year before staff reported data on COVID-19 infections. Additionally, using data provided by each of the Trusts, our sample was weighted to better represent the population from which they were drawn. Finally, our PPIE group helped to ensure that this study addressed issues pertinent to those experiencing the condition, e.g. by advising on what predictors we should include in our analyses, and shared their lived experiences which informed our interpretation of the results.

The results of our study must also be considered within some limitations. Firstly, data on sickness absence and on COVID-19 symptoms and their duration were self-reported. This may have influenced results, as these were not objective measures and may have been biased. For example, a minority of HCWs indicated that they did not take any sickness absence due to their COVID-19 infection, despite clinical guidance at both data collection timepoints mandating that all people with a COVID-19 infection complete an isolation period. This included clinical and non-clinical HCWs who could not reasonably work from home and should require time off work when infected with COVID-19. We cannot be certain if this is a biased measurement or if they are true estimates from HCWs who chose not to or were not allowed to take time off work. Secondly, we did not have an objective measure of whether HCWs required 4 + consecutive weeks off work at once. To overcome this, we approximated the number of HCWs who took long-term sickness absence using the total numbers of days off work and the total numbers of sickness episodes they reported. It may have been, however, that some HCWs who did take long-term sickness absence may not have been correctly categorised. Our sensitivity analysis, however, indicated that similar associations were present between our predictors and outcome when only including HCWs who reported one sickness episode.

Our research was not able to examine every factor which may have an impact on COVID-19-related sickness absence. For example, evidence suggests vaccination against COVID-19 significantly may reduce adult’s risk of developing LC [41], which in turn likely lowers their risk of requiring time off work. Similarly, studies suggest that infections related to the Omicron variant of the COVID-19 virus were associated with lower prevalence of LC and symptom severity compared to the Alpha and Delta variants, and that different variants are associated with different LC phenotypes [42], indicating that those infected by the Omicron variant may also require less sickness absence. While these variables are not available in NHS CHECK, we note that future studies should explore them, as they may interact or confound predictors that we have identified, as well as other unseen variables such as time since last vaccine, vaccine type (e.g. mRNA or other), or number of COVID-19 infections. A complete view of LC is important to understanding the mechanism(s) behind it.

Additionally, when looking for predictors of long-term sickness absence, we chose to analyse the data collected at 12 or 32 months separately given noticeable differences in reporting on sickness absence between both timepoints. This resulted in our regression model being under-powered [21], as evidenced by our wide confidence intervals. We recommend future studies to explore this in more detail with a sufficiently powered sample, which would allow for more robust findings. We also did not examine the severity of LC symptoms, and so are unable to examine if symptom severity was related with sickness absence, which previous research has explored [31]. Finally, while we offer an insight into absenteeism in our sample, we do not offer insights into other aspects of work participation, such as phased return to work and reasonable adjustments, presenteeism, or medical retirement due to LC. Future waves of NHS CHECK data collection will seek to include indicators on these aspects when examining the impact of COVID-19 and other illnesses on work participation.

## Conclusions

Our study found that prolonged COVID-19 symptoms are associated with increased sickness absence in HCWs in England. We found a noticeable reduction in the proportion of staff requiring long-term sickness absence due to COVID-19 infections between our two data collection time points (2021–2023). The reasons behind this are likely multi-faceted, but key drivers may include individual recovery, increased knowledge of LC and supports dedicated to affected staff, or the withdrawal of the special sickness absence payment available to NHS staff in September 2022. We acknowledge that LC is an episodic condition [43], and periodic symptom relapses may mean that people with the condition require sickness absence and/or workplace adjustments, even after first returning to work post-infection [31, 44]. Until further research leads to more specific treatments, the need to support and provide rehabilitation for those with LC remains.

## Supplementary Information

Below is the link to the electronic supplementary material.

Supplementary Material 1

## Data Availability

Due to Research Ethics Committee restrictions, the data set is not publicly available. Requests to access the deidentified data set can be made to the NHS CHECK team at [nhscheck@kcl.ac.uk](mailto: nhscheck@kcl.ac.uk) .

## Footnotes

1 Specifically, we had erroneously replaced the “More so than usual” response with “Not at all” for item 8.
