# Supplementary material for: COVID-19-related sickness absence among 4,721 NHS staff in England and its relation with long COVID symptoms: findings from NHS CHECK
Source: BMC Health Serv Res. 2025 Sep 30;25:1243. doi: 10.1186/s12913-025-13442-w (PMC12482608; doi:10.1186/s12913-025-13442-w)
Supplement: Supplementary file 1 — Supplementary Material 1 [file 12913_2025_13442_MOESM1_ESM.docx]

**Supplementary Table 1a.** Demographic and health information of the healthcare workers in NHS CHECK who completed the 12 month follow-up survey.

|  |  | **Total NHS CHECK sample** | **HCWs with a COVID-19 infection** | **Did not report LC symptoms** | **Reported LC symptoms** |
| --- | --- | --- | --- | --- | --- |
| **Variable** | **Categories** | ***n* = 24,137 (%)** | ***n* = 2,200 (%)** | ***n* = 1,288 (%)** | ***n* = 912 (%)** |
| Sex | Female | 19,381 (74.1) | 1,771 (75.2) | 997 (72.3) | 774 (80.8) |
|  | Male | 4,491 (24.9) | 416 (24.4) | 283 (27.3) | 133 (18.8) |
|  | Missing | 265 (1.0) | 13 (0.4) | 8 (0.4) | 5 (0.4) |
| Age in years | 30 and younger | 4,681 (21.3) | 370 (18.7) | 242 (21.6) | 128 (15.3) |
|  | 31–40 | 5,247 (25.0) | 446 (22.5) | 283 (23.2) | 163 (19.9) |
|  | 41–50 | 6,000 (22.0) | 593 (23.4) | 313 (21.5) | 280 (26.6) |
|  | 51–60 | 5,691 (20.7) | 612 (26.4) | 350 (25.0) | 262 (28.5) |
|  | 61 and older | 1,439 (6.5) | 99 (5.4) | 55 (5.5) | 44 (5.1) |
|  | Missing | 1,079 (4.5) | 80 (3.6) | 45 (3.2) | 35 (4.6) |
| Ethnicity | White | 20,507 (75.9) | 1,904 (79.6) | 1,101 (78.0) | 803 (82.1) |
|  | Black | 1,045 (8.0) | 82 (6.6) | 57 (7.8) | 25 (4.4) |
|  | Asian | 1,572 (12.1) | 132 (10.1) | 80 (10.2) | 52 (10.7) |
|  | Mixed/Other ethnicity | 817 (3.3) | 74 (3.4) | 45 (3.6) | 29 (2.6) |
|  | Missing | 196 (0.7) | 8 (0.3) | 5 (0.4) | 3 (0.2) |
| Relationship status | Single/Divorced | 6,304 (26.9) | 518 (23.0) | 304 (23.6) | 214 (23.3) |
|  | Married/Cohabitating | 17,592 (72.2) | 1,677 (76.9) | 982 (76.3) | 695 (76.5) |
|  | Missing | 241 (0.9) | 5 (0.1) | 2 (0.1) | 3 (0.2) |
| Probable CMDs* | No (GHQ-12 score <4) | 9,811 (40.9) | 778 (34.6) | 508 (39.6) | 270 (28.9) |
|  | Yes (GHQ-12 score ≥4) | 10,970 (45.9) | 978 (45.5) | 514 (39.4) | 464 (52.7) |
|  | Missing | 3,356 (13.2) | 444 (19.9) | 266 (21.0) | 178 (18.4) |
| Pre-existing respiratory illness* | None | 21,338 (89.1) | 1,741 (80.3) | 1,020 (79.6) | 721 (80.6) |
|  | Reported asthma/COPD | 1,216 (5.0) | 103 (4.3) | 42 (3.2) | 61 (6.3) |
|  | Missing | 1,583 (5.9) | 356 (15.4) | 226 (17.2) | 130 (13.1) |
| *n* = unweighted frequencies (with weighted percentages). NHS = National Health Service in the United Kingdom; HCWs = healthcare workers; LC = Long COVID (symptoms lasting for 12 weeks following acute COVID-19 infection); CMDs = common mental disorders; GHQ = General Health Questionnaire; COPD = Chronic Obstructive Pulmonary Disease.  *These variables were not asked to the replenishment sample, accounting for 356 missing responses from HCWs with a COVID-19 infection. | | | | | |

**Supplementary Table 1b.** Occupational information of the healthcare workers in NHS CHECK who completed the 12 month follow-up survey.

|  |  | **Total NHS CHECK sample** | **HCWs with a COVID-19 infection** | **Did not report LC symptoms** | **Reported LC symptoms** |
| --- | --- | --- | --- | --- | --- |
| **Variable** | **Categories** | ***n* = 24,137 (%)** | ***n* = 2,200 (%)** | ***n* = 1,288 (%)** | ***n* = 912 (%)** |
| Job role | Nurse | 6,127 (29.7) | 638 (32.8) | 340 (29.4) | 298 (38.1) |
|  | Doctor | 1,727 (9.8) | 171 (8.5) | 128 (10.8) | 43 (4.7) |
|  | Other clinical | 7,338 (32.1) | 720 (34.6) | 424 (35.2) | 296 (33.9) |
|  | Non-clinical | 8,744 (27.7) | 656 (23.6) | 387 (24.1) | 269 (22.7) |
|  | Missing | 201 (0.7) | 15 (0.5) | 9 (0.5) | 6 (0.6) |
| Perceived manager support* | No/A little support | 3,548 (17.3) | 316 (16.7) | 161 (14.3) | 155 (19.6) |
|  | Moderate/Extreme support | 18,158 (73.5) | 1,492 (66.4) | 885 (67.4) | 607 (65.5) |
|  | Missing | 2,431 (9.2) | 392 (16.9) | 242 (18.3) | 150 (14.9) |
| Perceived colleague support* | No/A little support | 1,697 (7.3) | 119 (5.1) | 62 (4.7) | 57 (5.5) |
|  | Moderate/Extreme support | 20,034 (83.6) | 1,691 (78.4) | 984 (77.3) | 707 (79.8) |
|  | Missing | 2,406 (9.1) | 390 (16.5) | 242 (18.0) | 148 (14.7) |
| Contract type | Permanent contract | 20,480 (85.3) | 1,928 (88.9) | 1,110 (87.7) | 818 (90.7) |
|  | Non-permanent contract | 3,386 (13.7) | 260 (10.7) | 172 (12.0) | 88 (8.8) |
|  | Missing | 271 (1.0) | 12 (0.4) | 6 (0.3) | 6 (0.5) |
| Thoughts on leaving the NHS | No | – | 370 (16.7) | 248 (19.3) | 122 (12.6) |
|  | Yes | – | 963 (44.5) | 515 (39.6) | 448 (50.6) |
|  | Missing | – | 867 (38.8) | 525 (41.1) | 342 (36.8) |
| Income | NHS Band 5 or below | 7,599 (27.7) | 691 (28.4) | 400 (28.2) | 291 (29.2) |
|  | NHS Band 6 or above | 12,570 (54.0) | 1,150 (55.6) | 646 (53.5) | 504 (58.4) |
|  | Missing | 3,968 (18.3) | 359 (16.0) | 242 (18.3) | 117 (12.4) |
| Contact with COVID-19 patients* | No contact | 8,200 (27.1) | 448 (16.3) | 310 (18.3) | 138 (13.3) |
|  | Contact | 10,309 (51.7) | 1,135 (57.3) | 608 (54.4) | 527 (61.1) |
|  | Missing | 5,628 (21.2) | 617 (26.4) | 370 (27.3) | 247 (25.6) |
| Perceived access to PPE* | Inadequate access | 1,709 (8.3) | 151 (8.0) | 74 (6.7) | 77 (9.2) |
|  | Adequate access | 17,130 (73.8) | 1,479 (68.6) | 861 (68.3) | 618 (69.7) |
|  | Non-applicable | 2,839 (8.6) | 169 (6.2) | 104 (6.3) | 65 (6.0) |
|  | Missing | 2,459 (9.3) | 401 (17.2) | 249 (18.7) | 152 (15.1) |
| Confidence in infection control * | Inadequate confidence | 8,211 (37.7) | 674 (33.6) | 367 (31.9) | 307 (35.6) |
|  | Adequate confidence | 13,406 (53.0) | 1,132 (49.5) | 671 (49.4) | 461 (49.9) |
|  | Missing | 2,520 (9.3) | 394 (16.9) | 250 (18.7) | 144 (14.5) |
| *n* = unweighted frequencies (with weighted percentages). NHS = National Health Service in the United Kingdom; HCWs = healthcare workers; LC = Long COVID (symptoms lasting for 12 weeks following acute COVID-19 infection); NHS Band 5 or below = Annual income of £34,581 or lower; NHS Band 6 or above = Annual income of £35,392 or higher (as of January 2024); PPE = personal protective equipment. Data for all variables (except reported thoughts about leaving the NHS) were collected at baseline, between April 2020 to January 2021, and will be used to explore risk factors for reporting long-term sickness absence. Reported thoughts about leaving the NHS were collected at 12 months.  *These variables were not asked to the replenishment sample, accounting for 356 missing responses from HCWs with a COVID-19 infection. | | | | | |

**Supplementary Table 2a.** Demographic and health information of the healthcare workers in NHS CHECK who completed the 32 month follow-up survey.

|  |  | **Total NHS CHECK sample** | **HCWs with a COVID-19 infection** | **Did not report LC symptoms** | **Reported LC symptoms** |
| --- | --- | --- | --- | --- | --- |
| **Variable** | **Categories** | ***n* = 24,137 (%)** | ***n* = 2,906 (%)** | ***n* = 2,122 (%)** | ***n* = 784 (%)** |
| Sex | Female | 19,381 (74.1) | 2,357 (74.4) | 1,703 (73.4) | 654 (77.4) |
|  | Male | 4,491 (24.9) | 534 (25.1) | 410 (26.1) | 124 (22.0) |
|  | Missing | 265 (1.0) | 15 (0.5) | 9 (0.5) | 6 (0.6) |
| Age in years | 30 and younger | 4,681 (21.3) | 313 (11.3) | 245 (12.6) | 68 (8.4) |
|  | 31–40 | 5,247 (25.0) | 543 (22.8) | 400 (23.1) | 143 (21.7) |
|  | 41–50 | 6,000 (22.0) | 865 (26.9) | 638 (27.1) | 227 (26.1) |
|  | 51–60 | 5,691 (20.7) | 875 (27.2) | 618 (25.9) | 257 (31.5) |
|  | 61 and older | 1,439 (6.5) | 202 (8.4) | 145 (8.1) | 57 (8.4) |
|  | Missing | 1,079 (4.5) | 108 (3.4) | 76 (3.2) | 32 (3.9) |
| Ethnicity | White | 20,507 (75.9) | 2,676 (85.0) | 1,947 (84.6) | 729 (86.6) |
|  | Black | 1,045 (8.0) | 53 (4.6) | 43 (4.7) | 10 (4.2) |
|  | Asian | 1,572 (12.1) | 86 (7.0) | 70 (7.4) | 16 (5.5) |
|  | Mixed/Other ethnicity | 817 (3.3) | 80 (3.1) | 55 (3.0) | 25 (3.3) |
|  | Missing | 196 (0.7) | 11 (0.3) | 7 (0.3) | 4 (0.4) |
| Relationship status | Single/Divorced | 6,304 (26.9) | 667 (24.1) | 490 (24.5) | 177 (26.1) |
|  | Married/Cohabitating | 17,592 (72.2) | 2,222 (75.4) | 1,621 (75.1) | 601 (75.3) |
|  | Missing | 241 (0.9) | 17 (0.5) | 11 (0.4) | 6 (0.6) |
| Probable CMDs* | No (GHQ-12 score <4) | 9,811 (40.9) | 1,143 (38.6) | 916 (43.5) | 227 (24.8) |
|  | Yes (GHQ-12 score ≥4) | 10,970 (45.9) | 1,360 (47.8) | 924 (43.5) | 436 (59.6) |
|  | Missing | 3,356 (13.2) | 403 (13.6) | 282 (12.9) | 121 (15.6) |
| Pre-existing respiratory illness* | None | 21,338 (89.1) | 2,460 (86.2) | 1,826 (87.8) | 634 (81.8) |
|  | Reported asthma/COPD | 1,216 (5.0) | 186 (5.5) | 115 (4.4) | 71 (8.3) |
|  | Missing | 1,583 (5.9) | 260 (8.3) | 181 (7.8) | 79 (9.9) |
| *n* = unweighted frequencies (with weighted percentages). NHS = National Health Service in the United Kingdom; HCWs = healthcare workers; LC = Long COVID (symptoms lasting for 12 weeks following acute COVID-19 infection); CMDs = common mental disorders; GHQ = General Health Questionnaire; COPD = Chronic Obstructive Pulmonary Disease.  *These variables were not asked to the replenishment sample, accounting for 260 missing responses from HCWs with a COVID-19 infection. | | | | | |

**Supplementary Table 2b.** Occupational information of the healthcare workers in NHS CHECK who completed the 32 month follow-up survey.

|  |  | **Total NHS CHECK sample** | **HCWs with a COVID-19 infection** | **Did not report LC symptoms** | **Reported LC symptoms** |
| --- | --- | --- | --- | --- | --- |
| **Variable** | **Categories** | ***n* = 24,137 (%)** | ***n* = 2,906 (%)** | ***n* = 2,122 (%)** | ***n* = 784 (%)** |
| Job role | Nurse | 6,127 (29.7) | 713 (29.9) | 491 (27.6) | 222 (32.8) |
|  | Doctor | 1,727 (9.8) | 171 (7.9) | 142 (8.9) | 29 (4.8) |
|  | Other clinical | 7,338 (32.1) | 857 (32.0) | 641 (32.7) | 216 (30.3) |
|  | Non-clinical | 8,744 (27.7) | 1,145 (30.5) | 832 (30.1) | 313 (31.5) |
|  | Missing | 201 (0.7) | 20 (0.7) | 16 (0.7) | 4 (0.6) |
| Perceived manager support* | No/A little support | 3,548 (17.3) | 416 (17.3) | 280 (16.3) | 136 (19.3) |
|  | Moderate/Extreme support | 18,158 (73.5) | 2,174 (72.4) | 1,618 (74.0) | 556 (68.7) |
|  | Missing | 2,431 (9.2) | 316 (10.3) | 224 (9.7) | 92 (12.0) |
| Perceived colleague support* | No/A little support | 1,697 (7.3) | 199 (7.9) | 129 (7.3) | 70 (9.2) |
|  | Moderate/Extreme support | 20,034 (83.6) | 2,393 (81.9) | 1,769 (83.0) | 624 (78.9) |
|  | Missing | 2,406 (9.1) | 314 (10.2) | 224 (9.7) | 90 (11.9) |
| Contract type | Permanent contract | 20,480 (85.3) | 2,600 (90.1) | 1,894 (90.2) | 706 (90.7) |
|  | Non-permanent contract | 3,386 (13.7) | 290 (9.3) | 215 (9.2) | 75 (8.8) |
|  | Missing | 271 (1.0) | 16 (0.6) | 13 (0.6) | 3 (0.5) |
| Thoughts on leaving the NHS | No | – | 694 (22.6) | 549 (23.9) | 145 (19.2) |
|  | Yes | – | 2,125 (74.2) | 1,511 (72.6) | 614 (78.3) |
|  | Missing | – | 87 (3.2) | 62 (3.5) | 25 (2.5) |
| Income | NHS Band 5 or below | 7,599 (27.7) | 855 (24.1) | 601 (22.9) | 254 (27.5) |
|  | NHS Band 6 or above | 12,570 (54.0) | 1,655 (60.7) | 1,226 (61.3) | 429 (59.4) |
|  | Missing | 3,968 (18.3) | 396 (15.2) | 295 (15.8) | 101 (13.1) |
| Contact with COVID-19 patients* | No contact | 8,200 (27.1) | 1,022 (26.9) | 804 (28.5) | 218 (22.4) |
|  | Contact | 10,309 (51.7) | 1,178 (50.4) | 838 (50.1) | 340 (50.9) |
|  | Missing | 5,628 (21.2) | 706 (20.7) | 480 (21.4) | 226 (26.7) |
| Perceived access to PPE* | Inadequate access | 1,709 (8.3) | 178 (6.8) | 118 (6.1) | 60 (8.7) |
|  | Adequate access | 17,130 (73.8) | 2,017 (71.5) | 1,490 (73.1) | 527 (67.2) |
|  | Non-applicable | 2,839 (8.6) | 380 (10.8) | 283 (10.5) | 97 (11.4) |
|  | Missing | 2,459 (9.3) | 331 (10.9) | 231 (10.3) | 100 (12.7) |
| Confidence in infection control * | Inadequate confidence | 8,211 (37.7) | 906 (33.6) | 644 (32.6) | 262 (35.5) |
|  | Adequate confidence | 13,406 (53.0) | 1,669 (55.8) | 1,243 (57.5) | 426 (51.9) |
|  | Missing | 2,520 (9.3) | 331 (10.6) | 235 (9.9) | 96 (12.6) |
| *n* = unweighted frequencies (with weighted percentages). NHS = National Health Service in the United Kingdom; HCWs = healthcare workers; LC = Long COVID (symptoms lasting for 12 weeks following acute COVID-19 infection); NHS Band 5 or below = Annual income of £34,581 or lower; NHS Band 6 or above = Annual income of £35,392 or higher (as of January 2024); PPE = personal protective equipment. Data for all variables (except reported thoughts about leaving the NHS) were collected at baseline, between April 2020 to January 2021, and will be used to explore risk factors for reporting long-term sickness absence. Reported thoughts about leaving the NHS were collected at 32 months.  *These variables were not asked to the replenishment sample, accounting for 260 missing responses from HCWs with a COVID-19 infection. | | | | | |

| **Supplementary Table 3.** Results from the multi-level logistic regression models exploring risk factors for long-term sickness absence among HCWs who reported Post COVID-19 Syndrome symptoms on the 12 and 32 month surveys and including only HCWs who reported one episode of sickness absence. | | | | | |
| --- | --- | --- | --- | --- | --- |
| **Variable** |  | **Odds of Long-Term Sickness Absence:** | | | |
|  |  | **at 12 months (*n* = 533)** | | **at 32 months (*n* = 496)** | |
|  | **Categories** | **aOR** | **95% CI** | **aOR** | **95% CI** |
| **Sex** | Female (Ref) | 1.00 | – | 1.00 | – |
|  | Male | 1.13 | [0.40, 3.19] | 3.55 | [0.61, 20.73] |
| **Age in years** | 30 and younger (Ref) | 1.00 | – | 1.00 | – |
|  | 31–40 | 7.54 | [0.90, 63.41] | 0.53 | [0.02, 13.93] |
|  | 41–50 | **5.09*** | [1.24, 20.87] | 2.23 | [0.25, 20.02] |
|  | 51–60 | **8.53**** | [2.01, 36.07] | 0.60 | [0.04, 8.28] |
|  | 61 and older | **8.91*** | [1.81, 43.85] | 4.03 | [0.27, 60.11] |
| **Ethnicity** | White (Ref) | 1.00 | – | 1.00^a^ | – |
|  | Black | 4.51 | [0.40, 51.40] | Empty | – |
|  | Asian | 1.27 | [0.62, 2.63] | Empty | – |
|  | Mixed/Multiple and Other ethnic group | 2.61 | [0.55, 12.38] | Empty | – |
| **Relationship status** | Single/Divorced (Ref) | 1.00 | – | 1.00 | – |
|  | Married/Cohabitating | 1.00 | [0.48, 2.11] | 3.27 | [0.26, 40.67] |
| **Probable common mental disorders** | No (GHQ-12 score <4; Ref) | 1.00 | – | 1.00 | – |
|  | Yes (GHQ-12 score ≥4) | 0.81 | [0.42, 1.57] | 0.85 | [0.11, 6.77] |
| **Pre-existing respiratory illness** | None (Ref) | 1.00 | – | 1.00 | – |
|  | Reported Asthma/COPD | **4.30**** | [1.63, 11.34] | **11.51*** | [1.11, 119.67] |
| **Job role** | Nurse (Ref) | 1.00 | – | 1.00 | – |
|  | Doctor | 0.56 | [0.16, 1.92] | 3.23 | [0.16, 65.08] |
|  | Other clinical | 0.45 | [0.18, 1.16] | 6.04 | [0.84, 43.40] |
|  | Non-clinical | **0.38**** | [0.19, 0.76] | 1.28 | [0.20, 8.23] |
| **Perceived manager support** | No/A little support (Ref) | 1.00 | – | 1.00 | – |
|  | Moderate/Extreme support | 0.56 | [0.15, 2.09] | 6.19 | [0.15, 247.35] |
| **Perceived collegial support** | No/A little support (Ref) | 1.00 | – | 1.00 | – |
|  | Moderate/Extreme support | 1.80 | [0.35, 9.22] | 0.31 | [0.02, 5.17] |
| **Contract type** | Permanent contract (Ref) | 1.00 | – | 1.00 | – |
|  | Non-permanent contract | 1.12 | [0.30, 4.17] | 0.03 | [0.01, 7.42] |
| **Reported intention to leave NHS** | No thoughts about leaving NHS (Ref) | 1.00 | – | 1.00 | – |
|  | Thought about leaving NHS | 0.93 | [0.39, 2.23] | 1.57 | [0.26, 9.67] |
| **Income** | NHS Band 5 or below (Ref) | 1.00 | – | 1.00 | – |
|  | NHS Band 6 or above | 0.77 | [0.29, 2.04] | 1.51 | [0.17, 13.78] |
| **Contact with COVID-19 patients** | No contact (Ref) | 1.00 | – | 1.00 | – |
|  | Contact | 0.82 | [0.27, 2.43] | 2.06 | [0.17, 24.26] |
| **Perceived access to personal protective equipment** | Perceived inadequate access (Ref) | 1.00 | – | 1.00 | – |
|  | Perceived adequate access | 0.78 | [0.21, 2.87] | 1.73 | [0.07, 43.85] |
|  | Non-applicable | 0.67 | [0.12, 3.63] | 3.02 | [0.03, 274.82] |
| **Confidence in infection control policies** | Inadequate confidence (Ref) | 1.00 | – | 1.00 | – |
|  | Adequate confidence | 0.73 | [0.35, 1.55] | 1.05 | [0.16, 7.03] |
| The 12 month survey was distributed between April 2021 to January 2022, while the 32-month survey was distributed between February to May 2023. HCWs = healthcare workers; long-term sickness absence = a period of four or more consecutive weeks off work attributed to a COVID-19 infection; aOR = Adjusted Odds Ratio, adjusted for all variables in the table, as well as burden on NHS when risk factor data was collected and Trust; 95% CI = 95% Confidence Intervals for the effect of each variable on the outcome. Due to small sample sizes and perfect prediction at 32 months, the Mixed/Multiple and Other ethnic groups were combined for analysis. **p* < 0.05; ***p* < 0.01. ^a^ No HCWs of non-white ethnicity who reported only one episode of sickness episode also reported long-term sickness absence. | | | | | |
